# APDM gait and balance measures fail to predict symptom progression rate in Parkinson's disease

**DOI:** 10.3389/fneur.2022.1041014

**Published:** 2022-11-09

**Authors:** D. Campbell Dewey, Shilpa Chitnis, Morgan C. McCreary, Ashley Gerald, Chadrick H. Dewey, Alexander Pantelyat, Ted M. Dawson, Liana S. Rosenthal, Richard B. Dewey

**Affiliations:** ^1^Department of Neurology, UT Southwestern Medical Center, O'Donnell Brain Institute, Dallas, TX, United States; ^2^Perot Foundation Neuroscience Translational Research Center, UT Southwestern Medical Center, O'Donnell Brain Institute, Dallas, TX, United States; ^3^Department of Neurology, Johns Hopkins University School of Medicine, Baltimore, MD, United States

**Keywords:** Parkinson's disease, gait, balance, disease progression, clinical trials

## Abstract

Parkinson's disease (PD) results in progressively worsening gait and balance dysfunction that can be measured using computerized devices. We utilized the longitudinal database of the Parkinson's Disease Biomarker Program to determine if baseline gait and balance measures predict future rates of symptom progression. We included 230, 222, 164, and 177 PD subjects with 6, 12, 18, and 24 months of follow-up, respectively, and we defined progression as worsening of the following clinical parameters: MDS-UPDRS total score, Montreal Cognitive Assessment, PDQ-39 mobility subscale, levodopa equivalent daily dose, Schwab and England score, and global composite outcome. We developed ridge regression models to independently estimate how each gait or balance measure, or combination of measures, predicted progression. The accuracy of each ridge regression model was calculated by cross-validation in which 90% of the data were used to estimate the ridge regression model which was then tested on the 10% of data left out. While the models modestly predicted change in outcomes at the 6-month follow-up visit (accuracy in the range of 66–71%) there was no change in the outcome variables during this short follow-up (median change in MDS-UPDRS total score = 0 and change in LEDD = 0). At follow-up periods of 12, 18, and 24 months, the models failed to predict change (accuracy in the held-out sets ranged from 42 to 60%). We conclude that this set of computerized gait and balance measures performed at baseline is unlikely to help predict future disease progression in PD. Research scientists must continue to search for progression predictors to enhance the performance of disease modifying clinical trials.

## Introduction

Parkinson's disease (PD) is associated with many putative environmental (1) and genetic (2) risk factors which in combination result in heterogeneity of onset symptoms, blend of motor and non-motor clinical features, and rate of disease progression. This disease heterogeneity has been recognized as a major challenge for disease modifying research because addressing mechanistic processes involved in one subtype would not be expected to slow progression of a different subtype. For example, some PD patients develop early dyskinesias and motor fluctuations suggesting acceleration of pathology in the nigral dopaminergic pathways while others develop early progression of gait difficulty and cognitive decline implying early spread of the alpha-synucleinopathy into the cerebral cortex. Accordingly, one of the highest priorities for clinical research identified by the National Institute for Neurological Disorders and Stroke (NINDS) Parkinson's 2014 Conference was to “characterize the long-term progression of PD and understand the mechanisms that underlie the heterogeneity in clinical presentation and rates of progression” (3). Moreover, the study of drugs aimed at slowing progression is limited by the practical need to organize clinical trials that can be completed in a reasonable time frame of < 5 years. The problem is that on average, PD progresses slowly with a median survival time of 15.8 years (4). Though the long range goal in pursuit of precision medicine is to design trials in well-defined, homogeneous biological subtypes, this will only be possible with future identification of robust prodromal biomarkers (5). In the meantime, being able to predict in early disease the likelihood that a given individual will progress rapidly could be useful for enriching disease modifying clinical trials with fast progressors.

PD results in significant gait and balance deterioration, features which are now amenable to objective assessment by computerized systems, and interest is developing in evaluating these features as possible biomarkers (6–9). Some studies have used pressure-sensitive walkways to objectively measure gait (10, 11), while other groups have employed high-resolution cameras (12, 13) or accelerometer-based approaches (14). Several reports have demonstrated that gait and balance features as measured by inertial sensors are useful for tracking progression in multiple sclerosis (15, 16). However, to date there has been insufficient study of instrumented gait and balance measures as potential longitudinal biomarkers of progression in PD.

The Parkinson's Disease Biomarker Program was established by the NINDS in 2012 to foster the prospective collection of clinical data and biological specimens in subjects with idiopathic Parkinson's disease followed longitudinally for up to 5 years (17, 18). In addition to collecting the NINDS Common Data Elements and biospecimens, we obtained instrumented gait and balance measurements using the APDM Mobility Lab (19) at two performance sites. We previously demonstrated that gait and balance parameters derived from this system can differentiate PD from control subjects and correlate with disease severity when assessed at a single time point (20). The aim of this exploratory analysis was to determine if gait and balance parameters measured at baseline could predict future rates of symptom progression as measured by various clinical outcomes.

## Materials and methods

### Standard protocol approvals, registrations, and patient consents

The study protocol was reviewed and approved by the UT Southwestern and Johns Hopkins School of Medicine Institutional Review Boards. Written informed consent was obtained from all participants. Though this study is not classified as a clinical trial and registration was therefore optional, we registered it on clinicaltrials.gov with registration number NCT01767818. The study is reported in accordance with STROBE guidelines for cohort studies (Supplementary Table 1).

### Subjects and experimental protocol

Detailed information on inclusion/exclusion criteria and the experimental protocol has been previously published (20). For this study, subjects with PD met UK brain bank criteria (21) and exhibited a favorable response to dopaminergic medication. Patients who were untreated with dopaminergic drugs at baseline were not included.

Participants were examined every 6 months for data collection that included the Movement Disorders Society revision of the Unified Parkinson's Disease Rating Scale (MDS-UPDRS) (22), Levodopa equivalent daily dose (LEDD) (23) as modified by incorporating the daily dose of carbidopa and levodopa extended-release capsules (Rytary) x 0.7 and carbidopa/levodopa enteral suspension (Duopa) x 0.97, biosample collection, and gait and balance testing with the APDM Mobility Lab (version 1) using two programs, the instrumented Timed Up and Go (iTUG) test (24) and the instrumented test of postural sway (iSway) (25). The iTUG calculates 96 unique variables describing gait, turns, sit to stand, and turn to sit transitions. The iSway produces 46 unique parameters describing the sway of the body in static stance. These tests were conducted by a study coordinator who read a standard instruction script to patients without additional prompts or encouragement. Test sessions were generally performed between 8 and 10 AM, and for PD fluctuators, all assessments were performed in the clinical “on” state. Subjects who arrived for testing in a clinical “off” state were given a dose of levodopa with testing delayed until he/she turned “on.” After affixing sensors to the wrists, ankles, lumbar area and chest, for the iTUG, patients stand from a chair, walk 6 meters, turn, walk back to the chair, and sit. For the iSway, patients stand for 30 s on a firm surface with their arms crossed and feet spaced 26 cm apart. The performance of these tests is demonstrated in the video clip (Supplementary Video 1). For each test program, subjects completed three trials, and the median value of each parameter was used for analysis. The entire gait and balance testing sequence took on average 10 min to complete per subject. Additionally, every 12 months the following instruments were obtained: Montreal Cognitive Assessment (MoCA) (26), Parkinson's disease questionnaire (PDQ-39) (27), and Schwab and England Activities of Daily Living Scale (S&E) (28).

During our longitudinal follow-up of study subjects, the APDM company released a new version of their hardware/software platform (version 2) which fundamentally changed how parameters are calculated from the raw sensor data. To enhance the value of this report for use by investigators in the future, we worked with the company to import our existing sensor data collected on version 1 of the platform into version 2 of the software and allowed it to generate version 2 parameters. Due to the change in hardware platform, this re-analysis of sensor data was only applicable to the iSway program. We thus also report results of our analysis of the version 2 re-calculation of the iSway sensor data. The reanalysis resulted in a total of 33 unique parameters for iSway version 2. Definitions for each program in the iTUG, iSway version 1 and iSway version 2 are presented in Supplementary Table 2.

For this analysis, we included subjects with at least two clinical assessments (baseline and one post-baseline visit) and at least one visit with gait or balance data from Mobility Lab. The first visit at which gait or balance data were available was deemed the baseline visit for this analysis. We classified subjects according to their follow-up duration. Subjects were classified in the 6-month group if they had baseline gait, balance, and clinical data and a clinical visit 6 months later. Grouping for 12, 18, and 24 months followed the same approach.

### Analytic plan

Because there is no uniformly accepted measurement of symptom progression in PD, we defined progression as the annual rate of change from baseline to 6, 12, 18, and 24 months of: MDS-UPDRS total score, MoCA, PDQ-39 mobility subscale, LEDD, S&E, and a global composite outcome (GCO) (29). The GCO is an equally weighted average of z-scores of MDS-UPDRS part I, MDS-UPDRS part II, MDS-UPDRS part III, S&E, and MoCA. Given that higher scores on the MDS-UPDRS subscales represent worse symptoms while the reverse is true of the S&E and MoCA, we inverted the sign of the z-scores corresponding to the S&E and MoCA to preserve equivalent directionality of the five measurements.

The analysis was designed to determine if any baseline gait or balance measurements could accurately predict symptom progression rate as measured by the clinical outcomes specified. To determine if any gait measures at baseline (i.e., iSway 1, iSway 2, or iTUG) or combinations of gait measures at baseline (i.e., iSway 1 + iTUG, iSway 2 + iTUG) can predict change in metrics of progression (MDS-UPDRS Total Score, LEDD, PDQ-39 Mobility Score, MoCA, Schwab and England, or GCO) at 6, 12, 18, and 24 months post-baseline, ridge regression models were estimated independently for each gait measure or combination of measures, metric of progression, and time from baseline. To determine the accuracy of each ridge regression model, cross-validation was performed in which 90% of the data were used to estimate the ridge regression model (i.e., training sample) and the corresponding model was used to estimate the rate of change in the corresponding metric of progression for a given time frame given the values of the gait measures (or combination of gait measures) in the 10% of data left out of the ridge regression model estimation (i.e., testing set). The measure of accuracy was chosen to be the concordance of the estimated rate of change and the true rate of change. That is, we measured the performance of the model to correctly order the subjects' rate of progression. We performed 10-fold cross validation with 1,000 replicates of 90% of subjects included in the training sample and the remaining 10% included in the testing set. We report both the average (standard deviation) in-sample accuracy, the concordance of predicted progression rates in the training set, and the average (standard deviation) out-of-sample accuracy, the concordance of predicted progression rates in the testing set. The out-of-sample accuracy is most representative of the performance of a model at predicting rate of change for future subjects.

All statistical analyses were carried out using R v. 4.2.0 (https://www.r-project.org/). Ridge regression was performed using the glmnet package in R (30). Accuracy of the model was calculated using Goodman and Kruskal's gamma. The formula is:


$$\begin{array}{l} y=\frac{C-D}{C+D} \end{array}$$

where C is number of concordant pairs, and D is the number of discordant pairs. When *y* = 1, perfect concordance exists.

## Results

Baseline clinical scores and demographic information were available for 230, 222, 164, and 177 PD subjects in the four follow-up groups as shown in Table 1. The groups were very similar with respect to clinical features. Table 2 shows the median change (in units per year) for the groups classified by duration of follow-up. Note that no significant change was seen at 6 and 12 months of follow-up in any clinical measure. By contrast, after 18 months of follow-up the MDS-UPDRS total score had increased by a median of 3 points with a 90 mg/day increase in LEDD. Further progression in all clinical measures was noted by 24 months of follow-up. The accuracy of APDM gait and balance measures at baseline for predicting future change in clinical outcomes is shown in Table 3. The results are poor out of sample accuracy for all time points except for modest prediction of progression at the 6-month follow-up visit. Of note, there was no actual change in the two clinical variables available at the 6-month time point.

**Table 1:** Demographic and baseline clinical measurements in subjects with the designated length of follow-up.

	**Subjects with**	**Subjects with**	**Subjects with**	**Subjects with**
	**6 months F/U**	**12 months F/U**	**18 months F/U**	**24 months F/U**
*N*	230	222	164	177
Male (%)	140 (60.87%)	138 (62.16%)	106 (64.63%)	104 (58.76%)
Hispanic (%)	18 (8.14%), *n* = 221	16 (7.48%), *n* = 214	15 (9.38%), *n* = 160	13 (7.69%), *n* = 169
Age	65.75 (34.75–85.58)	66.46 (34.75–85.58)	64.62 (34.75–85.58)	66.92 (34.75–85.58)
Years of education	16 (9–20), *n* = 228	16 (9–20), *n* = 220	16 (9–20), *n* = 162	16 (9–20), *n* = 175
Disease duration	4 (0–21)	4 (0–21)	4 (0–19)	4 (0–21)
**Baseline measurements**				
MDS-UPDRS				
Part I	8 (0–22)	8 (0–22)	7 (0–20)	8 (0–22)
Part II	8 (0–30)	8 (0–30)	7 (0–30)	7 (0–30)
Part III	17 (2–70)	17 (2–70)	16 (3–54)	17 (3–70)
Part IV	0 (0–20)	0 (0–20)	0 (0–20)	0 (0–20)
Total Score	36 (7–104)	36 (7–104)	32 (8–104)	35 (8–104)
Hoehn & Yahr	2 (1–4)	2 (1–4)	2 (1–4)	2 (1–4)
LEDD	600 (60–2,508.5)	600 (60–2,508.5)	563.75 (60–2,508.5)	600 (60–2,508.5)
S&E	0.9 (0.4–1)	0.9 (0.6–1)	0.9 (0.6–1)	0.9 (0.6–1)
MoCA	27 (17–31)	27 (17–30)	27.5 (17–30)	27 (17–30)
HAM-A	6.5 (0–29)	7 (0–29)	5 (0–27)	6 (0–29)
HAM-D	4 (0–23), *n* = 227	4 (0–23), *n* = 219	3.5 (0–23)	4 (0–23)
Epworth	7 (0–21)	7 (0–21)	6 (0–21)	7 (0–21)
UPSIT	18 (6–39), *n* = 228	18 (6–39), *n* = 219	18 (6–39), *n* = 163	18 (6–39), *n* = 174
GCO	−0.1 (−1.25 to 2.1)	−0.09 (−1.25 to 2.1)	−0.21 (−1.23 to 2.1)	−0.12 (−1.25 to 2.1)
PDQ-39 Mobility	5 (0–87.5), *n* = 229	7.5 (0–67.5), *n* = 221	5 (0–67.5), *n* = 163	6.25 (0–67.5), *n* = 176

MDS-UPDRS: Movement Disorders Society revision of the Unified Parkinson Disease Rating Scale; LEDD: Levodopa equivalent daily dose; S&E: Schwab and England Activities of Daily Living Scale; MoCA: Montreal Cognitive Assessment; HAM-A: Hamilton Anxiety Scales; HAM-D: Hamilton Depression Rating Scale; Epworth: Epworth Sleepiness Scale; UPSIT: University of Pennsylvania Smell Identification Test; GCO: Global Composite Outcome; PDQ-39 Mobility: Mobility subscale of the Parkinson's Disease Questionnaire. Cells containing “*n*=” denote the sample size of subjects with available data.

**Table 2:** Median change in each outcome variable by timepoint specified (units per year).

	**6-Month**	**12-Month**	**18-Month**	**24-Month**
Median MDS-UPDRS total score change (Q_1_, Q_2_)	0 (−6, 6)	0.5 (−6, 7.75)	3 (−5, 10)	5 (−3, 13)
Median LEDD change (Q_1_, Q_2_)	0 (0, 93.75)	0 (0, 199.5)	90 (0, 230.25)	119.5 (0, 350)
Median PDQ39 change (Q_1_, Q_2_)		0 (−2.5, 5)		2.5 (0, 10)
Median MoCA change (Q_1_, Q_2_)		0 (−1, 1)		−1 (−2, 1)
Median S&E change (Q_1_, Q_2_)		0 (−0.1, 0)		0 (−0.1, 0)
Median GCO change (Q_1_, Q_2_)		0.04 (−0.18, 0.28)		0.23 (−0.05, 0.53)

Q_1_: 25th percentile; Q_2_: 75th Percentile.

**Table 3:** Average in-sample (training set) and out-of-sample (testing set) accuracy (standard deviation) of ridge regression to predict change in six metrics from baseline to 6, 12, 18, and 24 months.

	**6-Month**		**12-Month**		**18-Month**		**24-Month**	
	**In-sample**	**Out-of-sample**	**In-sample**	**Out-of-sample**	**In-sample**	**Out-of-sample**	**In-sample**	**Out-of-sample**
**iSway 1**								
MDS-UPDRS Total score	76.23% (0.64%)	72.46% (6.53%)	56.14% (1.27%)	48.29% (7.37%)	62.86% (0.78%)	55.49% (7.3%)	66.02% (0.77%)	58.55% (8.4%)
LEDD	53.25% (1.16%)	45.53% (8.29%)	62.11% (0.86%)	53.43% (7.39%)	61.98% (0.75%)	56.65% (7.39%)	66.53% (0.77%)	58.75% (8.61%)
PDQ39			59.16% (0.94%)	54.98% (8.55%)			55.69% (1.24%)	46.77% (7.44%)
MoCA			54.02% (0.92%)	47.22% (8.27%)			54.06% (0.79%)	47.43% (6.84%)
Schwab			58.43% (1.36%)	51.52% (9.19%)			64.8% (0.83%)	60.05% (7.74%)
GCO			53.67% (0.81%)	49.61% (9.37%)			61.39% (0.72%)	54.48% (7.5%)
**iSway 2**								
MDS-UPDRS Total score	75.47% (0.65%)	71.17% (6.98%)	58.06% (0.82%)	52.35% (7.12%)	63.88% (0.73%)	57.47% (7.17%)	65.23% (0.79%)	59.4% (8.3%)
LEDD	52.27% (1.05%)	49.31% (8.75%)	60.91% (0.83%)	53.6% (7.52%)	60.49% (0.8%)	56.66% (7.64%)	64.97% (0.87%)	57.96% (8.64%)
PDQ39			60.83% (0.85%)	55.12% (8.74%)			55.1% (1.01%)	51.77% (7.82%)
MoCA			53.77% (1.16%)	42.76% (8.22%)			55.74% (0.81%)	48.12% (6.95%)
Schwab			61.39% (0.8%)	55.7% (8.87%)			63.93% (0.85%)	59.79% (7.93%)
GCO			53.04% (0.94%)	50.32% (9.41%)			59.92% (0.7%)	55.24% (7.45%)
**iTUG**								
MDS-UPDRS Total score	75.84% (0.65%)	67.64% (8.22%)	60.45% (1.16%)	51.55% (8.19%)	66.48% (0.78%)	56.08% (8.08%)	71.62% (0.83%)	58.21% (8.87%)
LEDD	57.75% (1.68%)	41.45% (8.31%)	58.57% (0.97%)	51.7% (8.29%)	62.95% (0.86%)	55.17% (8.35%)	63.04% (0.87%)	55.58% (8.82%)
PDQ39			64.06% (0.8%)	57.27% (9.19%)			58.56% (0.75%)	53.09% (7.87%)
MoCA			52.39% (2.42%)	43.36% (9.04%)			60.13% (0.87%)	52.97% (8.28%)
Schwab			59.25% (0.67%)	54.93% (9.17%)			69.17% (0.86%)	55.66% (7.82%)
GCO			50.59% (2.54%)	45.28% (9.8%)			70.43% (0.69%)	57.37% (7.9%)
**iSway 1 + iTUG**								
MDS-UPDRS Total score	76.04% (0.65%)	66.97% (7.91%)	60.86% (1.23%)	49.93% (8.21%)	68.89% (0.78%)	55.51% (8.14%)	73.95% (0.72%)	57.12% (9.23%)
LEDD	58.75% (1.45%)	42.43% (8.27%)	57.84% (1.21%)	49.89% (8.33%)	62.22% (0.86%)	54.41% (8.16%)	62.2% (0.84%)	54.61% (8.83%)
PDQ39			63.13% (0.82%)	56.93% (8.99%)			59.9% (1.05%)	52.14% (7.97%)
MoCA			53.45% (2.43%)	41.84% (8.79%)			58.5% (0.93%)	50.47% (8.42%)
Schwab			60.23% (0.74%)	53.83% (9.36%)			71.43% (0.72%)	56.4% (8.03%)
GCO			52.12% (2.02%)	47.23% (9.74%)			71.92% (0.67%)	58.1% (7.71%)
**iSway 2 + iTUG**								
MDS-UPDRS Total score	74.05% (0.62%)	66.74% (7.92%)	60.44% (1.3%)	51.55% (8.07%)	68.29% (0.73%)	56.28% (7.87%)	70.25% (0.71%)	57.19% (8.99%)
LEDD	57.94% (1.2%)	45% (8.47%)	58.57% (1.02%)	51.21% (8.17%)	62.7% (0.83%)	54.2% (8.19%)	61.88% (0.83%)	54.38% (8.6%)
PDQ39			63.55% (0.75%)	57.17% (8.87%)			60.72% (0.98%)	53.69% (8.24%)
MoCA			52.7% (2.38%)	41.34% (8.87%)			58.85% (0.92%)	51.22% (8.33%)
Schwab			61.15% (0.83%)	55.62% (9.4%)			57.12% (1.96%)	50.73% (8.66%)
GCO			51.96% (1.92%)	47.49% (9.79%)			69.01% (0.77%)	56.22% (8.31%)

## Discussion

Our results demonstrate that the APDM iSway (versions 1 and 2) and iTUG programs were not able to predict the future rate of symptom progression as measured by standard clinical outcomes at 12, 18, and 24 months of follow-up. The finding of modest prediction of progression as measured by MDS-UPDRS total score and LEDD in the out of sample group at 6 months is likely a true finding but with little significance because little or no change occurred in these parameters during this short time interval. Moreover, prediction of progression rate for only 6 months is not likely to be helpful for improving the efficiency of clinical trials of disease modifying agents. Such studies will typically need at least 2 years to show separation between an effective active drug and placebo because PD is a slowly progressive disease.

There are several limitations to our study. First, our analysis exhibited a high ratio of gait and balance parameters to the number of subjects; there were 147 instrumented parameters in our 230-patient cohort. This made our data subject to overfitting, which we addressed with the use of ridge regression. Second, while we controlled for baseline disease severity, levodopa-equivalent daily dose, and various demographic variables, there are many other factors which play a role in symptom progression that could not be controlled. For example, genetic factors are likely involved in disease progression rate as evidenced by the observation that patients with autosomal dominant SNCA triplication have faster progression as compared to those with recessively inherited PINK1 mutations (31). Third, we recognize that sensor-based gait and balance devices, such as the APDM Mobility Lab, have limitations that prevent a full and accurate characterization of human gait and balance. Among the most significant of these is that gait and balance are directly impacted by patient effort, and there is no way to control for variable effort at visits taking place months apart. Fourth, our study recruited subjects with mild to moderate PD with a median H&Y score of 2 and as such may not be representative of the larger population of PD. While we cannot exclude the possibility of Type-II error, our study likely included a sufficient number of subjects to detect a predictive ability of the APDM programs had this existed.

Strengths of this study include a sizable cohort of 230 medication-responsive PD patients with baseline and at least one follow-up visit, standardized rating of clinical variables, and follow-up of 2 years in most patients. Our statistical methods were carefully selected to ensure the lowest likelihood of reporting a false positive result, including multiple cross-validation analyses.

The many failed trials of putative neuroprotective agents in PD suggest that new approaches are needed to optimize clinical trial designs in ways that increase the probability of success. One promising approach is to enrich disease modifying trials with fast progressors who are more likely to show a difference between active drug and placebo within a reasonable amount of time. Development of novel outcome measures (including digitally-tracked measures for assessment at home and in the clinic) for PD clinical trials is warranted. While we are disappointed that the APDM programs failed to predict future progression, the search for ways to enrich PD clinical trials with fast progressors must continue.

## Data availability statement

Publicly available datasets were analyzed in this study. Demographic and clinical outcome data can be found here: https://pdbp.ninds.nih.gov/parkinsons-data. Additional information is available at article/Supplementary materials. APDM gait and balance data are available from the authors upon request.

## Ethics statement

The studies involving human participants were reviewed and approved by UT Southwestern and Johns Hopkins School of Medicine Institutional Review Boards. The patients/participants provided their written informed consent to participate in this study. Written informed consent was obtained from the individual for the publication of any potentially identifiable images or data included in this article.

## Author contributions

DD: study conception and first draft manuscript preparation. SC, LR, and RD: study design, analysis and interpretation of results, and manuscript editing. MM: data analysis, statistical design and computation, and statistical methods section writing. AG and CD: data collection and analysis. All authors reviewed the results, contributed to the interpretation of the data, revised it critically for important intellectual content, and provided final approval of the manuscript.

## Funding

This study was funded by NINDS U01-NS082148 and U01-NS082133 that supported the design and collection of data as part of the Parkinson's Disease Biomarker Program. The Jean Walter Center for Research in Movement Disorders supported a portion of the salary of the senior author.

## Conflict of interest

Author LR received the APDM second generation technology free of charge for use as a site-investigator in a research study in individuals with ataxia. Funding for that research comes from a collaboration between Pfizer, Biogen, and APDM, Inc. The remaining authors declare that the research was conducted in the absence of any commercial or financial relationships that could be construed as a potential conflict of interest.

## Publisher's note

All claims expressed in this article are solely those of the authors and do not necessarily represent those of their affiliated organizations, or those of the publisher, the editors and the reviewers. Any product that may be evaluated in this article, or claim that may be made by its manufacturer, is not guaranteed or endorsed by the publisher.
